# Readiness to change and therapy outcomes of an innovative psychotherapy program for surgical patients: results from a randomized controlled trial

**DOI:** 10.1186/s12888-017-1579-5

**Published:** 2017-12-29

**Authors:** Henning Krampe, Anna-Lena Salz, Léonie F. Kerper, Alexander Krannich, Tatjana Schnell, Klaus-Dieter Wernecke, Claudia D. Spies

**Affiliations:** 1 Department of Anesthesiology and Operative Intensive Care Medicine (CCM, CVK), Charité - Universitätsmedizin, corporate member of Freie Universität Berlin, Humboldt-Universität zu Berlin, and Berlin Institute of Health, Charitéplatz 1, 10117 Berlin, Germany; 2 Department of Anesthesiology, Intensive Care, Emergency and Pain Medicine, Hospital Wolfenbüttel gGmbH, Wolfenbüttel, Germany; 3 Department of Biostatistics, Clinical Research Unit, Berlin Institute of Health, Charité- Universitätsmedizin Berlin, Berlin, Germany; 4 Psychology of Personality and Individual Differences, Institute of Psychology, Leopold-Franzens-University, Innsbruck, Austria; 5 Institute of Medical Biometry, Campus Charité Mitte, Charité – Universitätsmedizin, Charitéplatz 1, 10117 Berlin, Germany; 6 SOSTANA GmbH, Berlin, Germany

**Keywords:** Cognitive behavioural therapy (CBT), Motivational interviewing (MI), Surgical patients, Bridging Intervention in Anesthesiology (BRIA), University of Rhode Island Change Assessment (URICA)

## Abstract

**Background:**

Readiness to change is a pivotal construct for psychotherapy research and a major target of motivational interventions. Our primary objective was to examine whether pre-treatment readiness to change moderated therapy effects of Bridging Intervention in Anesthesiology (BRIA), an innovative psychotherapy approach for surgical patients. This stepped care program aims at motivating and supporting surgical patients with mental disorders to engage in psychosocial mental health care.

**Methods:**

The major steps of BRIA are two motivational interventions with different intensity. The first step of the program consists of preoperative computer-assisted psychosocial self-assessment including screening for psychological distress and automatically composed computerized brief written advice (BWA). In the second step, patients participate in postoperative psychotherapy sessions combining motivational interviewing with cognitive behavioural therapy (BRIA psychotherapy sessions).

We performed regression-based moderator analyses on data from a recent randomized controlled trial published by our research group. The sample comprised 220 surgical patients with diverse comorbid mental disorders according to ICD-10. The most frequent disorders were mood, anxiety, substance use and adjustment disorders. The patients had a mean age of 43.31 years, and 60.90% were women. In a regression model adjusted for pre-treatment psychological distress, we investigated whether readiness to change moderated outcome differences between (1) the BRIA psychotherapy sessions and (2) no psychotherapy / BWA only.

**Results:**

Multiple regression analyses showed that readiness to change moderated treatment effects regarding the primary outcomes *"Participation in psychosocial mental health care options at month 6"* (*p* = 0.03) and *"Having approached psychosocial mental health care options at month 6"* (*p* = 0.048) but not regarding the secondary outcome *"Change of general psychological distress between baseline assessment and month 6"* (*p* = 0.329). Probing the moderation effect with the Johnson-Neyman technique revealed that BRIA psychotherapy sessions were superior to BWA in patients with low to moderate readiness, but not in those with high readiness.

**Conclusions:**

Readiness to change may act as moderator of the efficacy of psychosocial therapy. Combinations of motivational interviewing and cognitive behavioural therapy may be effective particularly in patients with a variety of mental disorders and low readiness to change.

**Trial registration:**

clinicaltrials.gov Identifier: NCT01357694.

## Background

The transtheoretical model of change (TTM) comprises five stages that people move through when they experience behaviour changes, pre-contemplation, contemplation, preparation, action, and maintenance, each of them contributing to the multifaceted construct of motivational readiness to change [1–4]. We applied this central component of the TTM to explore the relations between pre-treatment stages of change and treatment outcomes of Bridging Intervention in Anesthesiology (BRIA), an innovative transdiagnostic psychotherapy approach addressing surgical patients with comorbid mental disorders [5, 6]. This stepped care program aims at motivating and supporting patients from diverse surgical fields and with diverse mental disorders to engage in psychosocial mental health care options. BRIA combines different therapy elements of computer-assisted screening for psychological distress and brief intervention [7], motivational interviewing (MI) [8–10], cognitive behavioural therapy (CBT), and social casework (Table 1). The two major steps of BRIA are two motivational interventions with different intensity. The first step of the program consists of preoperative computer-assisted psychosocial self-assessment including screening for psychological distress and automatically composed computerized brief written advice (BWA). In the second step, patients participate in postoperative psychotherapy sessions combining MI and CBT (BRIA psychotherapy sessions). Whereas the BRIA psychotherapy sessions have a strong focus on MI, the computerized BWA can be considered as a weak motivational intervention because it does not involve therapy sessions but includes automatically composed detailed, individually tailored feedback of test results indicating psychological distress and, if required, general suggestions concerning therapy and behavior changes (Table 1). Readiness to change is a major target of motivational interventions [8, 9], and the question arises whether these two motivational interventions with different intensity show different efficacy in patients with varying degrees of motivation to change.

**Table 1:** Major therapeutic elements of the stepped care approach of BRIA (Bridging Intervention in Anesthesiology); Kerper et al., Effects of an innovative psychotherapy program for surgical patients - Bridging Intervention in Anesthesiology: A randomized controlled trial. Anesthesiology 2015;123:148-59 [6]

**Step I: Computer assisted psychosocial self-assessment and brief written computerized advice (BWA) for all patients**	
**Components** • Multiple-choice questions that can be answered by use of mouse, without requiring any input from keyboard • Duration: Approximately 25 min per patient • Items: Standardized psychological screening tests and single items concerning psychological distress, as well as social, lifestyle and psychological factors related to mental health and psychosocial therapy **Objective** • To offer computerized tailored BWA to all participants of the screening	**Topics of BWA** • *For patients without clinically significant psychological distress:* Positive feedback of screening test results indicating healthy lifestyle • *For patients with clinically significant psychological distress:* Detailed, individually tailored feedback of test results indicating psychological distress; if required suggestions concerning therapy and behavior changes • *Domains:* Depression, anxiety, general psychological distress, substance use problems (alcohol, illicit drugs, tobacco), well-being, quality of life, other health factors like weight, sleep and physical exercise
**Step II: BRIA psychotherapy sessions for patients with comorbid mental disorders**	
**Components** • Inpatient therapy sessions during hospital stay, outpatient sessions after discharge • Bedside visits and sessions in therapy rooms • Duration up to 50 min per session • Outpatient BRIA sessions up to 3 months postoperatively, booster sessions up to 6 months postoperatively **Objectives** • To motivate patients with mental disorders and support them in participating in subsequent psychosocial health care options • To initiate the improvement of patients’ psychological symptoms and well-being	**Topics of therapy sessions** • Detailed psychological assessment and clarification of diagnoses of mental disorders according to ICD-10 • Development of therapeutic alliance and activation of resources • MI to enhance motivation for therapy participation and behavior change • Emotional relief and individually oriented crisis interventions • Introduction of relaxation and stress management techniques • Guided discovery of reciprocal relationships between behavior, cognition, emotion and medical conditions • Elaboration of a biopsychosocial model of disease and health • Introduction to the concept of coping and problem skills training • Information on psychosocial mental health care options and teaching of skills how to apply for programs of psychosocial health care

Numerous psychotherapy studies have shown positive associations between higher motivation to change and treatment outcomes [3, 11–18]. Other studies, however, reported no, ambiguous or even negative associations [19–27]. The TTM aims to contribute to the development of interventions that are adapted to the patients’ readiness to change. Moreover, the stages of change have been conceived as being relevant across diverse problem areas and mental disorders [3, 28, 29]. Despite this basically transdiagnostic approach, the majority of psychotherapy research on stages of change has been carried out in the context of treatment of substance use disorders and eating disorders; far fewer studies exist concerning psychotherapy of other mental disorders [3, 13, 15, 16, 18, 30–32]. Theoretical models assume that motivation to change acts as a moderator of the efficacy of psychosocial therapy. However, previous treatment outcome research showed conflicting results [11, 12, 14, 15, 17, 20, 21, 23–25, 27, 32–41]. The findings of several studies suggest that motivational readiness to change is positively associated with pretreatment severity of problems and symptoms of those disorders for which patients seek therapy [21, 22, 26, 27, 30, 31]. Hence, we argue that the relations between readiness to change and psychotherapy outcomes may also be influenced by baseline psychological distress of patients.

This study aims to contribute to the clarification of the role of readiness to change in transdiagnostic therapy approaches by investigating its moderator effects on therapy outcomes of BRIA. A previous pilot study conducted by our research group showed feasibility of integrating BRIA into a surgical setting [5]. Afterwards we recently performed a randomized controlled trial (RCT) including 220 participants. In this RCT, BRIA psychotherapy sessions proved to be superior to the comparison intervention which did not include therapy sessions and consisted only of brief written computerized advice (BWA) based on patients’ results of computer-assisted screening for psychological distress in the first step of the program [6]. The primary outcome of the RCT was participation in psychosocial mental healthcare options at month 6, and the secondary outcome was change of self-reported general psychological distress between baseline and month 6, as measured with the Global Severity Index (GSI) of the questionnaire Brief Symptom Inventory (BSI [42, 43]) [6]. Concerning the primary outcome of the RCT, patients of the BRIA psychotherapy sessions group demonstrated a significantly increased engagement in subsequent psychosocial mental health care options at month 6 with an engagement rate of 30% compared with 11.8% in BWA only, resulting in a relative risk reduction of 0.21 and a number needed to treat of 6. Participants of BRIA psychotherapy sessions and BWA did not differ regarding the categories of psychosocial mental health care that they engaged in. The most frequent category was psychotherapy (52.2%), followed by psychosocial counselling (41.3%), self-help groups (4.3%), and psychiatric treatment (2.2%) [6]. Concerning the secondary outcome, BRIA psychotherapy sessions had a small but significant effect on the improvement of psychological distress, as measured with the BSI-GSI, between baseline and 6-month follow-up. To summarize, first results suggest feasibility and efficacy of BRIA [5, 6]. As an important next step, moderator analyses should follow.

This study addresses key questions concerning the relations between readiness to change, pre-treatment psychological distress and therapy outcomes of the 220 participants of the RCT of BRIA. Our primary objective was to examine whether pre-treatment readiness to change might moderate the outcome differences between BRIA psychotherapy sessions and BWA when analyses were adjusted for pre-treatment psychological distress.

## Method

### Setting and study design

In this study, a-priori planned moderator analyses are performed on data from a recent randomized, parallel-group, controlled trial which was conducted in the preoperative assessment clinics of the university hospital Charité – Universitätsmedizin Berlin, Campus Charité Mitte and Campus Virchow Klinikum, Berlin, Germany [6]. This RCT was approved by the hospital’s Ethics Committee [EA1/014/11] and was registered with clinicaltrials.gov. All patients provided written informed consent.

Inclusion criteria: Patients of the preoperative anesthesiological assessment clinic; age ≥ 18 years; acute significant mental distress, i.e. scoring above of at least one of the cut-off points of a set of six scales or subscales of four established psychological screening tests covering depression, anxiety, well-being, and alcohol use disorders (Table 2), and/or being tobacco smoker, and/or having consumed illicit substances during the last year. Exclusion criteria: Surgery with an urgent or emergency indication; inability to visit the preoperative assessment clinic; insufficient knowledge of German language; hospital staff; admitted in police custody; accommodation in an institution by official or court order; being under guardianship; psychiatric, neurological or other condition associated with limited legal capability or limited capability of being properly instructed or giving informed consent; severe acute mental disorder (acute episode of psychotic disorder, severe substance use disorder including serious withdrawal symptoms), severe acute suicidal ideation; homelessness; participating in psychosocial or substance use disorder treatment, participation in a psychopharmacological clinical trial at baseline assessment or 1 month before, respectively. Patients with acute severe psychiatric conditions were offered immediate crisis interventions because randomization was considered as unethical.

**Table 2:** Measures of the randomized controlled trial of BRIA; Kerper et al., Effects of an innovative psychotherapy program for surgical patients - Bridging Intervention in Anesthesiology: A randomized controlled trial. Anesthesiology 2015;123:148-59 [6]

Computer assisted preoperative self-assessment to measure clinically significant psychological distress and psychosocial factors
*World Health Organization 5-item Well-Being Index (WHO-5)*: Short depression / well-being screening tool of the WHO. Time frame: Past 14 days. Scaling: 5 items, 6-point scale from 0 to 5; total score from 0 to 25; Cut-off: WHO-5 sum score < 14 [51].
*Patient Health Questionnaire-4 (PHQ-4)*: Ultra-brief screening tool with subscales for depression (PHQ-2), and generalized anxiety (GAD-2). Time frame: Past 14 days. Scaling: 5 items, 4-point scale from 0 to 3; Sum scores of PHQ-2 and GAD-2 ranging from 0 to 6, with cut-off’s for both subscales of ≥3 [52].
*Hospital Anxiety and Depression Scale (HADS)*: Short screening tool with subscales for depression (HADS-D) and anxiety (HADS-A). Time frame: Past 7 days. Scaling: 14 items, 4-point scale from 0 to 3; for HADS-D and HADS-A each 7 items. Sum scores ranging from 0 to 21 with cut-off’s for HADS-D ≥ 9, HADS-A ≥ 11 [53].
*Alcohol Use Disorder Identification Test (AUDIT)*: WHO screening tool for hazardous and harmful alcohol consumption, and alcohol-related problems. Time frame: Past 12 months. Scaling: 10 items, 5-point scale from 0 to 4; total score from 0 to 40. Cut-off: AUDIT sum score: ≥8 for men; ≥5 for women [54].
*Single items:* Tobacco smoking, use of illicit drugs, loneliness, sleeping disturbance, physical activity, weight / height, pain, use of psychoactive medication, participation in psychosocial / addiction therapy.
**Postoperative questionnaires, interviews and medical scores to measure clinical characteristics, outcomes and moderator variables**
*Semistructured interview including the Short Diagnostic Interview for Mental Disorders (MiniDIPS)*: Diagnoses of mental disorders according to ICD-10; anxiety, mood, adjustment, trauma-related, obsessive-compulsive, somatoform, eating, and substance use disorders; screening questions for psychotic disorders [44].
*University of Rhode Island Change Assessment (URICA)*: Self-report questionnaire measuring motivational readiness to change according to the transtheoretical model of change. Time frame: Current state. Scaling: 16 items, 5-point scale from 1 to 5; composite measure of readiness to change from −2 to 14 [62, 63].
*Brief Symptom Inventory (* *BSI): *Self-report questionnaire measuring diverse symptoms of psychological distress. Time frame: Past 7 days. Scaling: 53 items, 5-point scale from 0 to 4. Total mean score Global Severity Index (GSI) from 0 to 4 [42, 43].
*Semistructured telephone interview assessing 2 measures of therapy engagement:* (1) Participation in psychosocial mental health care, defined as undergoing, being on a waiting list or having completed a psychosocial mental health care program other than BRIA itself during the 6 months after inclusion in the trial. (2) Having approached a psychosocial mental health care program regardless of whether this approach resulted in a participation in therapy sessions or a place on a waiting list.
*Medical measures:* (1) Preoperative physical health status according to the ASA (American Society of Anesthesiologists) physical status classification system [46, 47]; (2) Surgical field: neuro-, head and neck surgery; abdomino-thoracic surgery; peripheral surgery [5, 65–67]; (3) Severity of medical comorbidity according to the Charlson Comorbidity Index (CCI) [48]; (4) Extent of the specific surgical procedures according to the 4-point item ‘operative severity’ of the POSSUM scoring system (Physiological and Operative Severity Score for the enUmeration of Mortality and Morbidity) [49, 50].

Table 1 shows the stepped care approach of BRIA. It consists of two major steps, (1) a preoperative computer-assisted psychosocial self-assessment including BWA for all patients of the preoperative anesthesiological assessment clinics, and (2) postoperative BRIA psychotherapy sessions for patients with comorbid mental disorders. In the first days after surgery, study psychotherapist visited those participants of the preoperative self-assessment who fulfilled the eligibility criteria and who were interested to participate in the RCT. After re-assessment of the eligibility criteria and provision of written informed consent to participate in the RCT, the patients completed baseline postoperative psychological questionnaire and clinical diagnostic interview assessment. The study psychotherapists evaluated clinical characteristics in a semistructured clinical interview and made diagnoses of mental disorders according to ICD-10. They used the Short Diagnostic Interview for Mental Disorders (MiniDIPS [44]), a German extended adaptation of the Anxiety Disorders Interview Schedule [45]. The MiniDIPS covers anxiety, obsessive-compulsive, trauma-related, mood, adjustment, somatoform, eating, as well as substance use disorders. Additionally, there are screening questions for psychotic disorders. When the interview results suggested disorders that were not covered sufficiently by the MiniDIPS, therapists assessed the symptoms as well as time and impairment criteria by free exploration and made adequate diagnoses according to the ICD-10, e.g. in case of personality disorders or psychological and behavioural factors associated with disorders or diseases classified elsewhere (ICD F54). After having completed postoperative baseline diagnostic procedures, the patients were randomly allocated in a 1:1 ratio to receive one of the two study interventions: (1) The BRIA psychotherapy sessions (*n* = 110); (2) no additional intervention after the BWA (n = 110). The full details of the setting, patient recruitment, randomization, concealment of allocation and blinding, psychological and medical measurements, sample size calculation, data collection and analyses, as well as results of primary analyses are available in our previous publication [6].

### Patients

**Fig. 1:**
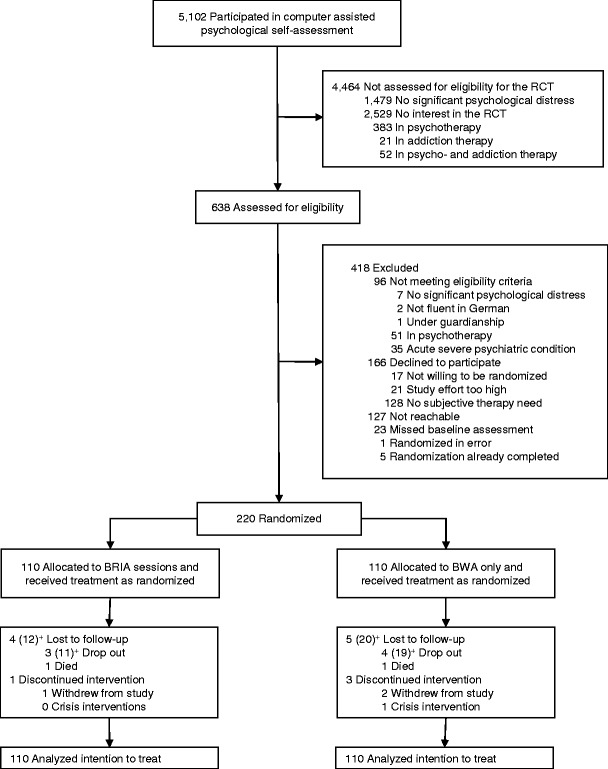
Flow of participants through the trial. ^+^ Numbers in brackets refer to patients lost to follow-up and drop outs concerning the secondary outcome. Numbers of patients lost to follow-up differ between primary and secondary outcomes because the primary outcome was assessed via a telephone interview, and the secondary outcome as a postal questionnaire. Kerper et al., Effects of an innovative psychotherapy program for surgical patients - Bridging Intervention in Anesthesiology: A randomized controlled trial. Anesthesiology 2015;123:148-59 [6]

In total, 220 patients from diverse surgical fields and with diverse mental disorders were included (Fig. 1). As shown in Table 3, the intervention groups did not differ significantly regarding demographic and clinical baseline characteristics. The mean BSI-GSI score indicating general psychological distress was 0.86 in both groups, and the most frequent mental disorders were mood, anxiety, substance use, and adjustment disorders.

**Table 3:** Demographic and clinical characteristics; n (%), mean [SD]; Kerper et al., Effects of an innovative psychotherapy program for surgical patients - Bridging Intervention in Anesthesiology: A randomized controlled trial. Anesthesiology 2015;123:148-59 [6]

	BRIA sessions *n* = 110		BWA only *n* = 110		***p***
Age (Years)	44.83	[13.66]	41.80	[13.01]	0.094
Women	69	(62.7)	65	(59.1)	0.679
General psychological distress: BSI-GSI	0.86	[0.53]	0.86	[0.55]	0.986
Readiness to change: URICA sum score	6.55	[2.18]	6.24	[2.26]	0.306
*Primary diagnosis of mental disorder*					
Emotional Disorders	91	(82.7)	87	(79.1)	
Mood disorder	37	(33.6)	40	(36.4)	
Anxiety disorder	31	(28.2)	26	(23.6)	
Adjustment disorder	19	(17.3)	13	(11.8)	
Somatoform disorder	1	(0.9)	4	(3.6)	
Eating disorder	1	(0.9)	2	(1.8)	
Personality disorder	1	(0.9)	2	(1.8)	
Psychological factors associated with diseases classified elsewhere ^a)^	1	(0.9)	0	(0.0)	
Substance use disorders	19	(17.3)	23	(20.9)	
Tobacco use disorder	12	(10.9)	12	(10.9)	
Alcohol use disorder	5	(4.5)	10	(9.1)	
Illicit substance use disorder	1	(0.9)	0	(0.0)	
Multiple substance use disorder	1	(0.9)	1	(0.9)	0.675 ^b)^
*Living together status*					
Living with a partner, married	43	(39.1)	29	(26.4)	
Living with a partner, not married	19	(17.3)	20	(18.2)	
Not living with a partner	48	(43.6)	61	(55.5)	0.110
*Employment status*					
Employed	73	(66.4)	67	(60.9)	
Unemployed	12	(10.9)	12	(10.9)	
Pension / Invalidity pension	12	(10.9)	11	(10.0)	
Undergoing education / training	6	(5.5)	12	(10.9)	
Residual group ^c)^	7	(6.4)	8	(7.3)	0.676
*Physical health (ASA Classification)*					
ASA I	29	(26.4)	28	(25.5)	
ASA II	67	(60.9)	71	(64.5)	
ASA III	14	(12.7)	11	(10.0)	
ASA IV	–	–	–	–	0.811
*Surgical field*					
Neuro-, head and neck surgery	22	(20.0)	32	(29.1)	
Abdomino-thoracic surgery	51	(46.4)	37	(33.6)	
Peripheral surgery	37	(33.6)	41	(37.3)	0.117
*Medical comorbidity (CCI)*					
0 ‘None’	74	(67.3)	76	(69.1)	
1 ‘Low’	19	(17.3)	19	(17.3)	
2 ‘Moderate’	5	(4.5)	9	(8.2)	
3 ‘High’	12	(10.9)	6	(5.5)	0.366
*Extent of surgical procedure* (POSSUM operative severity item)					
1 ‘Minor’	34	(30.9)	33	(30.0)	
2 ‘Moderate’	35	(31.8)	34	(30.9)	
4 ‘Major’	26	(23.6)	32	(29.1)	
8 ‘Major +’	15	(13.6)	11	(10.0)	0.735
Hospital length of stay	5.78	[7.03]	5.77	[8.74]	0.993

^a)^Psychological and behavioural factors associated with disorders or diseases classified elsewhere (ICD F54);^b)^ *p* refers to the comparison of the 2 intervention groups regarding the distribution of all of the 11 psychiatric diagnoses that were made; ^c)^ Working at home, gap year, not specified. *ASA* American Society of Anesthesiologists physical status classification: (I) Healthy patient; (II) mild systemic disease, no functional limitation; (III) severe systemic disease with definite functional limitation; (IV) severe systemic disease that is a constant threat to life; BSI-GSI = Brief Symptom Inventory, total score General Severity Index (range: 0-4); *CCI* Charlson Comorbidity Index, *POSSUM* Physiological and Operative Severity Score for the enUmeration of Mortality and Morbidity; item operative severity; URICA sum score: University of Rhode Island Change Assessment composite score (range: −2 to 14)

Concerning preoperative physical health and medical comorbidity, the majority of the patients was evaluated as healthy or having mild systemic disease according to the American Society of Anesthesiologists physical status classification (ASA) [46, 47], and as having no or low comorbidity according to the Charlson Comorbidity Index (CCI) [48]. Participation in the RCT was not restricted to any specific surgeries, and there was a slightly lower percentage of patients being treated in neuro-, head and neck surgery, as compared to abdomino-thoracic and peripheral surgery. The majority of surgical procedures was minor and moderate, while about 40% of surgeries were evaluated as major or major plus according to the operative severity item of the Physiological and Operative Severity Score for the enUmeration of Mortality and Morbidity (POSSUM) [49, 50].

### Interventions and therapists

#### Brief written computerized advice (BWA)

Surgical patients undergoing preoperative clinical examination by an anesthesiologist participated in a preoperative computer-assisted psychosocial self-assessment. Immediately after the completion of the self-assessment, the patients’ data were analyzed automatically and all patients received a computerized, individually tailored detailed feedback on their screening results in the form of a computer printout. This intervention was computerized and was not performed by psychotherapists. The BWA included for patients with elevated psychological distress general suggestions concerning therapy and behavior changes (Table 1). The screening tests comprised the questionnaires World Health Organization 5-item Well-Being Index (WHO-5, [51]), Patient Health Questionnaire-4 (PHQ-4, [52]), Hospital Anxiety and Depression Scale (HADS, [53]), Alcohol Use Disorder Identification Test (AUDIT, [54]), as well as several single-item questions concerning sociodemographic, lifestyle and clinical characteristics which are available from the authors upon reasonable request (Table 2). The BWA was programmed as web application with a technology using the server scripting language php and the database system MySQL.

Patients received two printouts, a short written letter and a page with a table of their individual questionnaire scores and information on the applied questionnaires and cut-off values. The automatically composed sections of the BWA letter were based on the individual self-assessment responses of the patients, and the text was explicitly formulated in a careful and considerate way in order to prevent inducing resistance and anger. In the main part of the letter, the individual symptoms were described and explained. Patients with scores above the cut-off were informed that their current elevated distress could be temporary and might decrease by itself. However, in case that it would persist for a longer period of time, patients were advised to consult their general practitioner to get to know and discuss possible treatment options. Finally, they were encouraged to show the table with their test results in potential appointments with health care professionals.

#### BRIA psychotherapy sessions

Major therapeutic elements of the stepped care approach of BRIA are displayed in Table 1. Whereas patients in the BWA group received only the screening and brief written advice intervention according to step 1, the BRIA psychotherapy group had the possibility to participate in psychotherapy sessions described in step 2. BRIA sessions were provided by a team of certified psychologists during inpatient hospital stay as well as in an outpatient setting, either face-to-face or by telephone, for a period of up to 3 months after discharge, with the possibility of booster sessions up to 6 months, and a maximum number of 12 sessions per patient. The intervention was based on the principles and methods of MI described by Miller & Rollnick (2002) [8] and Arkowitz et al. (2008) [9]. Therapists explored the patients’ experience and perspectives concerning their mental disorders, psychological distress, and problematic behavior, as well as ambivalence concerning appropriate target behavior change. The topics included motivation to engage in subsequent therapy options, as well as change of individual problematic behavior, e.g. behavioural activation in mood disorders or substance use reduction in substance use disorders. Emphasis was placed on a client-centred way of counselling, the development of therapeutic alliance, expressing empathy and explicit activation of patients’ resources, capacities and self-efficacy. The treatment concept described therapy practice according to the overall spirit of MI in terms of acceptance, recognizing the patients’ autonomy and self-direction, partnership, evocation and compassion. MI-specific directive strategies and methods were used to elicit and strengthen change talk, to prevent and decrease sustain talk, to support self-efficacy and to roll with resistance. Therapists switched from MI to more action-oriented CBT methods when patients’ responses signalized that they had decided to search for therapy options and wanted to use the BRIA sessions to start first behavior changes until the subsequent therapy would begin. In this phase, therapists used diverse established manuals for CBT of specific disorders, e.g. standard manuals for depressive disorders [55], anxiety disorders [56–58], and substance use disorders [59–61]. They chose basic interventions that they considered as adequate for the patients’ specific disorders, and that would be easy to integrate in subsequent therapies. Examples of these interventions are: emotional relief and individually oriented crisis interventions; relaxation and stress management techniques; guided discovery of reciprocal relationships between behavior, cognition, emotion and medical conditions; psychoeducation and elaboration of a biopsychosocial model of disease and health; functional analyses; self-monitoring; activity scheduling; as well as basic coping and problem skills training. For patients who had decided to search for subsequent therapy, CBT included also psychoeducation and information on psychosocial mental health care options and teaching and training of skills how to apply for treatment programs. Therapists practiced the CBT methods also in a general way of the spirit of MI, so that a switch back to explicit and specific MI strategies was feasible in times when patients experienced again increased ambivalence. The general procedures of the stepped care approach are documented in the unpublished study protocol, which is part of the application for approval by the ethics committee. A document describing the general procedures is available from the authors upon reasonable request.

The duration of a therapy session could be determined by the individual needs of the patients and rarely exceeded 50 min. The median number of therapy sessions per patient was 3 with an interquartile range (IQR) of 1 to 7. The median cumulative time spent in therapy sessions amounted to 83.5 min per patient (IQR: 28.8 – 285.5), and the median duration of a session was 27.3 min (IQR: 12.3 – 52.1). Out of all sessions, 16% were face-to-face during inpatient hospital stay, 56% were face-to-face in an outpatient setting, 27% were by telephone and 1% was done by email.

#### Therapists

A team of psychologists certified by diploma delivered the BRIA sessions. Throughout the recruitment period, the team consisted permanently of two to three therapists and one supervising therapist. The total number of therapists was seven, and the percentage of patients per therapist ranged from 2.7% to 26.4%, with a median of 13.6%. They had a median age of 29 years (25 years to 42 years) and six of them were women. Five therapists were participants of an advanced training program for CBT, and one therapist participated in an advanced training program for hypnotherapy. The supervising therapist had practiced MI since more than 10 years, had a licence for CBT for 7 years, as well as experience in teaching and training of MI for psychotherapists, medical health care professionals and medical students for more than 6 years. The six therapists in training had a median of 27.5 months of practical work experience in clinical psychology (10 months to 68 months), and a median of 17.25 months of practical experience in psychotherapy (10 months to 32 months). They had applied to an advertisement that described BRIA as a combination of MI and CBT. In the job interview, potential therapists were introduced to the BRIA concept and asked to reflect whether they felt like practicing a client-centred directive method of therapy. The training took about 1 week. It consisted of a workshop, reviewing of therapy videos, reading, as well as role-play with feedback from the supervisor and advanced therapists. Throughout the recruitment period, there was a weekly team meeting of approximately 2 h during which therapists and the supervising therapist discussed relevant issues of all current patients. Furthermore, therapists had approximately 1 weekly individual supervision session. During both the training and the supervision sessions, the therapists and the supervisor discussed the process of the sessions, the goals of patients and therapists, the application of MI and/or CBT strategies and methods, and how to practice a communication style in terms of the general spirit of MI.

During their practical work, therapists used the basic textbook of MI of Miller & Rollnick (2002) [8], as well as the pivotal textbook edited by Arkowitz et al. (2008) which introduced the approach to apply MI in combination with CBT in emotional disorders and psychological problems other than substance use problems [9]. They also used the unpublished study protocol (including a table that listed different categories of combinations of disorders and patient goals and the according therapy strategies), a comprehensive handout of the training workshop (including acronyms like OARS [Open Question, Affirm, Reflect, Summary]), a five-page handout of specific MI interventions, as well as established CBT manuals for diverse mental disorders, e.g. depression [55], anxiety disorders [56–58], and substance use disorders [59–61]. Concerning the combination of MI and CBT, therapists were encouraged to put the major focus on motivational interventions and to combine them with CBT interventions as described above.

### Measures

#### Motivational readiness to change

Readiness to change was measured at postoperative baseline assessment with a German short version of the University of Rhode Island Change Assessment (URICA) [62, 63]. The URICA consists of four subscales measuring the change stages pre-contemplation, contemplation, action, and maintenance; the preparation scale was eliminated during the development of the URICA because its items loaded too high on the components measuring contemplation and action [28, 29]. The 16-item short version is a self-report scale with 4 items for each of the four subscales. Items are rated on a 5-point scale from 1 to 5, with higher scores indicating greater endorsement of attitudes reflecting the respective stage of change. A composite measure of readiness to change is calculated by subtracting the precontemplation subscore from the sum of the subscores of contemplation, action, and maintenance, and it ranges from −2 to 14 [4]. This readiness to change score can also be expressed as a URICA total sum score, which includes the 12 item scores of contemplation, action, and maintenance, as well as the four inverted item scores of the pre-contemplation scale. This procedure allows for calculating internal consistency, which resulted in a good Cronbach’s alpha of 0.82 in the present study.

#### Therapy engagement

The original primary outcome of the RCT assessed the participation of patients in psychosocial mental health care options 6 months after inclusion in the study. Participation in psychosocial mental health care was defined as undergoing, being on a waiting list or having completed a psychosocial mental health care program other than BRIA itself during the 6 months after inclusion in the trial, e.g. psychotherapy, psychiatric treatment, psychosocial counselling, substance use treatment including counselling and smoking cessation services, as well as self-help groups. A second primary outcome was defined for this study as having approached psychosocial mental health care options regardless of whether this approach resulted in a participation in therapy sessions or a place on a waiting list.

Therapy engagement was assessed by research assistants via a semistructured telephone interview. Before their first interview, evaluators were trained in the application of the interview guideline, and during the 16 months of assessment, the evaluators held regular calibration meetings with supervising researchers. A major topic of the interview training and the calibration meetings was the use of measures to prevent response bias and to detect false positive answers. Concerning therapy engagement, the evaluators asked the patients to describe the treatment options that they said they had been engaging in. Then evaluators asked further questions to distinguish treatment by psychosocial health care professionals (e.g. psychological counselling, psychotherapy, psychiatry) from treatment by other health care professionals that comprised psychological topics (e.g. talking about one’s depression with a primary care physician). Research assistants were unaware of treatment assignment in order to guarantee rater blinding. At the beginning of the interview, evaluators told the patients that they were not involved in the therapy process, and they explicitly asked the patients not to reveal treatment assignment. During the telephone interviews, several patients reported that they had contacted therapists or psychosocial health care institutions but were neither put on a waiting list nor invited for a first session. Reasons for this were, for example, that staff asked patients to call once more at a later time point because even waiting lists were overbooked, or that they used answering machines without calling the patient back. In order to take account of these first activities of patients’ therapy engagement, a second primary outcome was calculated which consists of both participation in mental health care options and having approached psychosocial mental health care options without having managed to be put on a waiting list or to receive a first session. This alternative outcome reflects treatment engagement from a less strict perspective because it also includes those patients who might have participated in therapy if mental health care programs were more easily accessible.

#### General self-reported psychological distress

We measured general psychological distress with the total mean score Global Severity Index (GSI) of the Brief Symptom Inventory (BSI-GSI) [42, 43]. The BSI is a 53-item short form of the SCL-90-R (Symptom Checklist 90-R), an established and validated self-report scale of psychological distress. The BSI has proven feasibility in patients with medical conditions and it has sound psychometric properties in both patient and community samples [42, 43]. The items of the BSI refer to severity of various psychological symptoms during the past 7 days and they are rated on a 5-point scale from 0 (not at all) to 4 (extremely). The GSI is calculated by dividing the sum of item scores by the number of completed items. It has shown validity as a measure of general psychological distress by reflecting both the number and intensity of symptoms of perceived mental distress [42, 64]. At postoperative baseline assessment, patients completed the BSI as a paper-and-pencil questionnaire in the first days after surgery, and at 6-month follow-up, as a paper-pencil-questionnaire sent by post. In this study, the BSI showed good reliability with Cronbach’s alphas of 0.95 at baseline and 0.96 at 6-month follow-up.

#### Medical measures

The preoperative physical health status was assessed by the evaluation of patients’ perioperative risk according to the ASA (American Society of Anesthesiologists) physical status classification system [46, 47]. The surgical field encompassed the categories 1) neuro-, head and neck surgery, 2) abdomino-thoracic surgery, as well as 3) peripheral surgery [5, 65–67]. The severity of medical comorbidity was measured with the Charlson Comorbidity Index (CCI, [48]), a validated weighted classification system of comorbidity to measure cumulative burden of disease. We assessed the extent of the specific surgical procedures that patients underwent with the 4-point item ‘operative severity’ of the POSSUM scoring system (Physiological and Operative Severity Score for the enUmeration of Mortality and Morbidity) [49, 50].

### Statistical analyses

Descriptive results were expressed as relative frequencies in percent, as well as means and standard deviations. All analyses are based on the intention-to-treat data set of the primary analyses of the RCT of BRIA [6]. Comparisons of the treatment groups were performed with Fisher’s exact test for categorical data, and with t-test for continuous data. For all statistical tests, a two-tailed *p*-value ≤0.05 was considered statistically significant.

Moderation analyses were conducted using the PROCESS macro [68, 69] for SPSS, version 23 [70]. Multiple regression models tested whether the independent variables treatment assignment to BRIA psychotherapy sessions versus BWA, the continuous readiness-to-change composite score of the URICA, the interaction between treatment and readiness to change, as well as the baseline GSI-BSI score had statistically significant associations with three major treatment outcome variables. Multiple binary logistic regression models were calculated for the analyses concerning the two outcome variables that were binary: the primary endpoint of the RCT, ‘Participation in psychosocial mental health care options at month 6’, and an alternative version of the primary outcome, ‘Having approached psychosocial mental health care options at month 6’. A multiple linear regression model was calculated for the analyses concerning the outcome variable that was continuous: the secondary outcome of the RCT, ‘Change of self-reported general psychological distress, measured by the BSI-GSI, between baseline assessment and month 6’. The statistical interaction between the independent variables ‘Treatment assignment’ and ‘Readiness to change’ indicated whether the outcome differences between treatment groups were moderated by readiness to change. In order to probe the interactions, analyses using the Johnson-Neyman technique were conducted for all three outcome variables [69]. The Johnson-Neyman technique calculates the statistical significance of the effect of an independent variable, in this study treatment allocation, for all values of the moderator variable, in this study readiness to change. Thus, the Johnson-Neyman technique can ‘identify points of transition along the continuum of the moderator between a statistically significant and nonsignificant effect of X’ ([69], page 13). The resulting ranges of the values of the moderator where the independent variable is significantly associated with the dependent variable are called regions of significance.

## Results

Pretreatment readiness to change was not associated to treatment allocation, age, gender, diagnosis of mental disorder, as well as medical and perioperative characteristics. However, it was related to pre-treatment general psychological distress with a correlation between the readiness to change score and the BSI-GSI of *r* = 0.429 (*p* < 0.001).

Table 4 shows the results of multiple regression models analyzing the prediction of therapy outcomes at month 6. The models are adjusted for treatment allocation, motivational readiness to change, the interaction between treatment and readiness, as well as pre-treatment BSI-GSI. Treatment allocation, readiness to change, and the interaction between them had statistically significant independent effects on ‘Participation in psychosocial mental health care options’ and ‘Having approached psychosocial mental health care options at month 6’. However, they did not show significant effects concerning the outcome ‘Change of the BSI-GSI between baseline assessment and month 6’, which was only significantly predicted by the pretreatment BSI-GSI.

**Table 4:** Prediction of therapy outcomes at month 6 by treatment allocation, motivational readiness to change, and baseline GSI-BSI; *N* = 220 surgical patients

	Participation in psychosocial mental health care options			Having approached psychosocial mental health care options			Decrease of the BSI-GSI		
	Coefficient (SE)	Z	*p*	Coefficient (SE)	Z	*p*	Coefficient (SE)	t	*p*
Treatment allocation ^a)^	3.76 (1.30)	2.89	0.004	3.40 (1.14)	2.98	0.003	0.23 (0.16)	1.50	0.135
Readiness-to-change score	0.36 (0.15)	2.45	0.015	0.38 (0.13)	2.88	0.004	0.02 (0.02)	1.23	0.219
Treatment allocation x readiness-to-change score	−0.37 (0.17)	−2.17	0.030	−0.31 (0.16)	−1.98	0.048	−0.02 (0.02)	−0.98	0.329
Pretreatment GSI	0.08 (0.35)	0.23	0.820	0.32 (0.31)	1.02	0.307	0.19 (0.05)	3.68	<0.001

^a)^0 = BWA, 1 = BRIA

**Fig. 2:**
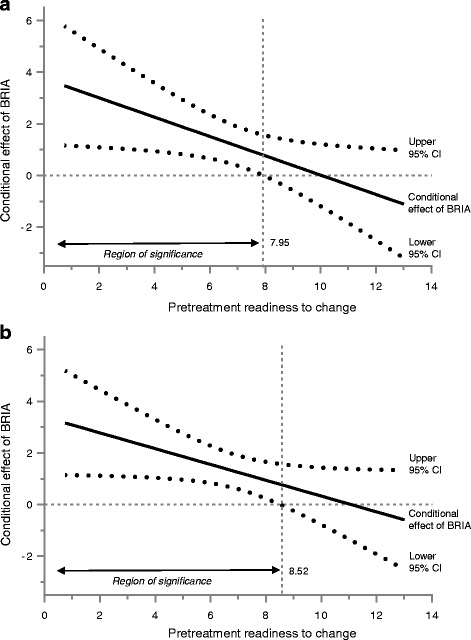
Johnson-Neyman plots of the interaction between treatment allocation (BRIA psychotherapy sessions vs. BWA, N = 220) and readiness to change. **a** Participation in psychosocial mental health care options at month 6; **b** Having approached psychosocial mental health care options at month 6. The black continuous lines show the conditional effects of BRIA psychotherapy sessions for all values of readiness to change, and the dotted lines above and below indicate the corresponding 95% confidence intervals (CI). The gray vertical dashed lines identify the regions of significance, reaching from the lowest score until the score of readiness to change at which the lower 95% CI of the slope crosses the zero point. For all readiness values below, the effect of BRIA psychotherapy sessions is statistically significant

The major results concern the two engagement measures. They indicate that BRIA psychotherapy sessions were superior to BWA, that higher readiness to change was related to better outcomes, and that the treatment effect of BRIA psychotherapy sessions was moderated by readiness to change. Analyses employing the Johnson-Neyman technique revealed that the conditional effect of BRIA psychotherapy sessions was significant for participants with low to moderate readiness to change scores, but not for those with high scores (Fig. 2a, b). Concerning ‘Participation in psychosocial mental health care options’, the region of significance ranged from the lowest readiness score to the value of 7.95 and covered 77.73% of the sample (*p*-values from 0.0004 to 0.05). For ‘Having approached psychosocial mental health care options at month 6’, the region of significance ranged from the lowest readiness score to the value of 8.52 and covered 85.45% of the sample (p-values from <0.0001 to 0.05). For the outcome ‘Change of the BSI-GSI between baseline assessment and month 6’, the Johnson-Neyman technique did not identify any region of significance.

## Discussion

The moderator analyses of this study established differential associations between readiness to change and therapy outcomes of Bridging Intervention in Anesthesiology. This transdiagnostic psychotherapy program combines elements of MI and CBT in order to motivate and support surgical patients with diverse mental disorders to engage in psychosocial mental health care options. The major results suggest that pretreatment readiness to change moderates the treatment effects of the two interventions BRIA psychotherapy sessions and BWA concerning the primary outcomes ‘Participation in psychosocial mental health care options’ and ‘Having approached psychosocial mental health care options’, but that there is no moderator effect of readiness to change concerning the secondary outcome ‘Change of general psychological distress’.

Readiness to change showed significant conditional effects in the prediction of the outcomes ‘Participation in psychosocial mental health care options’ and ‘Having approached psychosocial mental health options’, and it moderated the treatment effects of BRIA. These findings are consistent with previous research showing positive associations between higher motivation to change and treatment outcomes of psychotherapy [3, 11–18]. Like in previous studies readiness was positively associated with pretreatment psychological distress [21, 22, 26, 27, 30, 31]. This result emphasizes the importance to include baseline distress as a covariate in the moderator analyses of readiness to change. However, readiness did not have a significant association with the decrease of psychological distress, confirming previous findings indicating that higher readiness to change is not a unique predictor of therapy success [19–27]. Interestingly, pre-treatment psychological distress was not significantly related with participating in or having approached psychosocial mental health care options, but it was positively associated with decrease of distress after 6 months. This finding might partially be influenced by the methodological issue that patients with a higher level of baseline distress have a higher range of potential decrease. The relationship between severity of baseline symptoms and therapy results seems indeed not to be unequivocal since psychotherapy research has reported relationships between severity of symptoms and both poor and good treatment response [71].

Previous analyses of randomized controlled trials reported contradictory results concerning the moderating effects of readiness to change. Several trials did not find interactions between motivation to change and treatment assignment [15, 20, 21, 23, 25, 27, 38, 39]. Whereas some studies reported contradictory results of moderator analyses [12, 35, 37, 41], other trials showed that different active interventions worked differently successful in patients with low or high readiness [34, 36]. There is also evidence that patients with pain and high readiness have better outcomes in CBT compared to standard treatment [32]. Additionally, a recent investigation of students who smoke marijuana showed that among those with high readiness, a substance-specific web-based feedback intervention was more successful than general health related feedback [24]. Finally, there are data from three RCT’s of addiction therapy that found moderating effects with better outcomes among patients with low readiness to change, whereas among those with high readiness, the study interventions did not differ from each other [17, 33, 40]. Barnett et al. (2010) investigated young adult problem drinkers in the emergency department and found in those with low readiness to change better drinking outcomes at 12-months follow-up after motivational interviewing versus feedback only [33]. The results of the Project MATCH Group (1997) showed that among alcohol dependent patients with low readiness to change, motivational enhancement therapy yielded better drinking outcomes at 15-month follow-up than CBT [40]. In the RCT of Pantalon et al. (2002), patients with concurrent alcohol and cocaine use disorders and low readiness had higher percentage of days of substance abstinence when assigned to psychotherapy plus disulfiram medication as compared to psychotherapy only [17]. Several factors may contribute to the equivocal empirical status concerning the moderating effects of readiness to change on the efficacy of psychosocial therapy. On the one hand, the cited studies are rather heterogeneous regarding sample sizes, problem severity of participants, inclusion of additional moderating variables, type and number of outcomes, as well as type, intensity and duration of experimental and comparison interventions. On the other hand, most trials are from the addiction field, and moderator analyses of readiness to change in the treatment of other mental disorders are sparse. In addition, methodological issues might play a role like lack of statistical power, artificial results due to multiple testing, overlooking of other important potential moderators, and under- or overfitting of regression models.

Moderator analyses of the study at hand resulted in findings that are comparable to the results of Barnett et al. (2010) [33], the Project MATCH Group (1997) [40] and Pantalon et al. (2002) [17]. The data revealed that BRIA psychotherapy sessions yielded significantly better results in patients with low to moderate readiness to change concerning the engagement outcome measures. On the other hand, BRIA sessions were not significantly superior to BWA in patients with readiness scores above 7.95 and 8.52, respectively, which are noticeably higher than the mean readiness score of 6.40 (SD 2.22) of the total sample and can be interpreted as indicating high readiness to change. Altogether, these findings add to the research on the relations between motivation to change and psychotherapy by suggesting that interventions like the BRIA psychotherapy sessions that combine MI and CBT may especially be effective in those with low readiness to change. Importantly, the efficacy of motivational interventions might particularly affect outcomes that can be interpreted as possible achievements of increased motivation, like active search for therapy and engagement in therapy.

### Limitations and future directions

The data of this study are based on the first RCT comparing BRIA psychotherapy sessions with a weak comparison group, BWA. In order to guarantee treatment integrity, we took important steps during selection, training and supervision of therapists, as well as concerning the provision of therapy material for the therapists. However, we did not assess integrity and therapy processes with measures of therapist and client verbal language and nonverbal behavior during therapy, and we did not collect data on client motivation at follow-up. Therefore, it could not be investigated to which extent participation in BRIA sessions might have been related to an increase of the patients’ motivation to change and readiness for subsequent psychotherapy. Moreover, there are no data on the extent to which the therapists balanced interventions of MI and CBT during the specific BRIA sessions. Consequently, it remains unclear which specific components of BRIA contributed to improve patients’ active search for therapy and engagement in therapy, and which components were, in turn, more related to a decrease of psychological distress.

The moderator analyses of this study could not be adjusted for therapist allocation, because the BWA patients were not allocated to therapists. Since BWA was a computerized intervention without therapists, it is rather unlikely that the moderator effect of readiness to change on treatment allocation is due to a therapist effect. However, we cannot rule out that the findings might have been different with a more homogeneous or heterogeneous therapist team. From a very strict perspective, one might even argue that the effect of the BRIA psychotherapy sessions might only be due to the fact that it was performed by therapists whereas BWA was a computerized intervention. Indeed our data cannot reveal which ingredients of BRIA contribute to which extent to its therapy effect. Moreover, until now, there is no validated therapist-delivered face-to-face intervention for this specific setting with which the BRIA psychotherapy sessions could have been compared. Independent from the question why BRIA sessions are superior to BWA, the current moderator analyses identified an interesting interaction effect showing that patients who are less ready to change profit remarkably from a more intensive intervention including face-to-face contacts, MI and basic elements of CBT. In patients with high readiness, however, the therapy outcomes of BRIA sessions and BWA do not differ from each other.

It has to be mentioned that the primary engagement outcomes, participation in or having contacted psychosocial mental health care options, were based on self-report data since we could not collect objective data because of professional discretion and data protection. In order to ensure the validity of the outcome, the assessment of therapy engagement was done by means of a semi-structured interview including an interview booklet, training of evaluators, calibration sessions, blinding of evaluators and also measures to prevent response bias. Furthermore, it can be assumed that there was no reason for patients to answer in terms of social desirability, because they did not receive any reward for answering in one specific direction.

Whereas the inclusion of patients with diverse mental disorders can be seen as an example for the feasibility of transdiagnostic psychotherapy programs, it is also related to methodological limitations concerning the measurement of psychological symptoms. The BSI was chosen as major secondary outcome measure in order to have a symptom scale that would be applicable to assess general psychological distress of all patients. Given the relatively small GSI-BSI outcome differences between BRIA psychotherapy sessions and BWA that were found in the primary analyses of this study [6], the question arises of whether the GSI-BSI was suitable to measure change of symptoms in a sample that consisted of small subsamples of patients with heterogeneous disorders and the corresponding symptoms.

A formal limitation of this study is that the data sets are not publicly available because there is no approval of the study participants.

Taken together, future studies that aim to investigate the relations between motivation to change and combinations of MI and CBT should assess the behavior of therapists with appropriate observational instruments, e.g. the Motivational Interviewing Skills Code (MISC; [72]), the Motivational Interviewing Treatment Integrity Code (MITI 4; [73]), or the Cognitive Therapy Rating Scale (CTRS; [74]). It is also advisable to perform repeated measurements of patients’ motivation to change by analyses of within session language during therapy and by self-report questionnaires at follow-up. Finally, the inclusion of patients with homogeneous groups of disorders would facilitate the assessment of therapists’ application of specific CBT interventions.

## Conclusions

Previous studies of interactions between motivation and treatment allocation were mainly based on therapy research of substance use disorders. This investigation included surgical patients with diverse mental disorders, and the results suggest that combinations of MI and CBT are particularly effective in patients with low to moderate readiness to change. These data contribute to recent research showing that integrating MI with CBT is a promising therapy approach for patients with a variety of psychological problems that are not related to substance use disorders [75–77]. The results of patients with high readiness to change are difficult to interpret. Since they did not achieve significantly better outcomes in BRIA sessions compared with BWA, the question arises whether low-intensity interventions might be sufficient for patients with high readiness to change. An alternative interpretation suggests that BRIA sessions might not have contained enough specific CBT elements to treat patients with high readiness to change who might not have profited significantly from MI because their motivation had already been increased before the therapy. In order to investigate this topic in more detail, future research could analyze interactions between readiness to change and treatment allocation to low-intensity interventions like BWA, or to high-intensity MI-CBT combinations focusing either on MI or on CBT interventions.

Overall, the findings of this study suggest that motivation to change may act as a moderator of the efficacy of psychosocial therapy and that combinations of MI and CBT are particularly effective in patients with a variety of mental disorders and with low readiness to change. This efficacy seems to be especially observable in proximal outcomes that are assumed to be associated with motivation, like active search for therapy and engagement in therapy.

## Abbreviations

ASA: American Society of Anesthesiologists
AUDIT: Alcohol Use Disorder Identification Test
BRIA: Bridging Intervention in Anesthesiology
BWA: Brief written advice
BSI: Brief Symptom Inventory
BSI-GSI: Global Severity Index (GSI) of the Brief Symptom Inventory
CBT: cognitive-behavioural therapy
CCI: Charlson Comorbidity Index
CTRS: Cognitive Therapy Rating Scale
GSI: Global Severity Index
HADS: Hospital Anxiety and Depression Scale
IQR: Interquartile range
MI: Motivational Interviewing
MiniDIPS: Diagnostisches Kurz-Interview bei psychischen Störungen [Short diagnostic interview for mental disorders]
MISC: Motivational Interviewing Skills Code
MITI 4: Motivational Interviewing Treatment Integrity Code 4
PHQ-4: Patient Health Questionnaire-4
POSSUM: Physiological and Operative Severity Score for the enUmeration of Mortality and Morbidity
RCT: Randomized controlled trial
SCL-90-R: Symptom Checklist 90-R
TTM: Transtheoretical model of change
URICA: University of Rhode Island Change Assessment
WHO-5: World Health Organization 5-item Well-Being Index

## References

[1] Prochaska JO, DiClemente CC (1983). Stages and processes of self-change of smoking: toward an integrative model of change. J Consult Clin Psychol.

[2] Prochaska JO, DiClemente CC, Norcross JC (1992). In search of how people change. Am Psychol.

[3] Norcross J, Krebs P, Prochaska J (2011). Stages of change. J Clin Psychol.

[4] DiClemente CC, Carbonari J, Zweben A, Morrel T, Lee R, Longabaugh R, Wirtz P (2001). Motivation hypothesis causal chain analysis. Project MATCH: hypotheses, results, and causal chain analyses.

[5] Lange LF, Spies CD, Weiß-Gerlach E, Neumann T, Salz A-L, Tafelski S, Hein J, Seiferth N, Heinz A, Glaesmer H, Brähler E, Krampe H (2011). Bridging intervention in Anaesthesiology: first results on treatment need, demand and utilization of an innovative psychotherapy program for surgical patients. Clin Health Promot.

[6] Kerper LF, Spies CD, Salz A-L, Weiß-Gerlach E, Balzer F, Neumann T, Tafelski S, Lau A, Neuner B, Romanczuk-Seiferth N, Glaesmer H, Wernecke K, Brähler E, Krampe H (2015). Effects of an innovative psychotherapy program for surgical patients - bridging intervention in anesthesiology: a randomized controlled trial. Anesthesiology.

[7] Neumann T, Neuner B, Weiss-Gerlach E, Tønnesen H, Gentilello LM, Wernecke K-D, Schmidt K, Schroeder T, Wauer H, Heinz A, Mann K, Mueller JM, Haas N, Kox WJ, Spies CD (2006). The effect of computerized tailored brief advice on at-risk drinking in subcritically injured trauma patients. J Trauma.

[8] Miller WR, Rollnick S (2002). Motivational interviewing. Preparing people for change, 2nd edition.

[9] Arkowitz H, Westra HA, Miller W, Rollnick S (2008). Motivational interviewing in the treatment of psychological problems.

[10] Moyers TB, Miller W (2013). Is low therapist empathy toxic?. Psychol Addict Behav.

[11] Aharonovich E, Stohl M, Ellis J, Amrhein P, Hasin D (2014). Commitment strength, alcohol dependence and HealthCall participation: effects on drinking reduction in HIV patients. Drug Alcohol Depend.

[12] Capone C, Wood M (2009). Thinking about drinking: need for cognition and readiness to change moderate the effects of brief alcohol interventions. Psychol Addict Behav.

[13] Dray J, Wade T (2012). Is the transtheoretical model and motivational interviewing approach applicable to the treatment of eating disorders? A review. Clin Psychol Rev.

[14] Gaume J, Bertholet N, Daeppen J-B (2017). Readiness to change predicts drinking: findings from 12-month follow-up of alcohol use disorder outpatients. Alcohol Alcohol.

[15] Lewis CC, Simons AD, Silva SG, Rohde P, Small DM, Murakami JL, High RR, March JS (2009). The role of readiness to change in response to treatment of adolescent depression. J Consult Clin Psychol.

[16] Mander J, Wittorf A, Teufel M, Schlarb A, Hautzinger M, Zipfel S, Sammet I (2012). Patients with depression, somatoform disorders, and eating disorders on the stages of change: validation of a short version of the URICA. Psychotherapy.

[17] Pantalon MV, Nich C, Frankforter T, Carroll KM (2002). The URICA as a measure of motivation to change among treatment-seeking individuals with concurrent alcohol and cocaine problems. Psychol Addict Behav.

[18] Soler J, Trujols J, Pascual J, Portella M, Barrachina J, Campins J, Tejedor R, Alvarez E, Pérez V (2008). Stages of change in dialectical behaviour therapy for borderline personality disorder. Br J Clin Psychol.

[19] Abd Elbaky GB, Hay PJ, le Grange D, Lacey H, Crosby RD, Touyz S (2014). Pre-treatment predictors of attrition in a randomised controlled trial of psychological therapy for severe and enduring anorexia nervosa. BMC Psychiatry.

[20] Arnaud N, Baldus C, Elgán TH, Tønnesen H, De Paepe N, Csemy L, Thomasius R (2015). Moderators of outcome in a web-based substance use intervention for adolescents. Sucht.

[21] Field C, Adinoff B, Harris T, Ball S, Carroll K (2009). Construct, concurrent and predictive validity of the URICA: data from two multi-site clinical trials. Drug Alcohol Depend.

[22] Myers B, van der Westhuizen C, Naledi T, Stein DJ, Sorsdahl K (2016). Readiness to change is a predictor of reduced substance use involvement: findings from a randomized controlled trial of patients attending south African emergency departments. BMC Psychiatry.

[23] Nosyk B, Geller J, Guh DP, Oviedo-Joekes E, Brissette S, Marsh DC, Schechter MT, Anis AH (2010). The effect of motivational status on treatment outcome in the north American opiate medication initiative (NAOMI) study. Drug Alcohol Depend.

[24] Palfai TP, Tahaney K, Winter M, Saitz R (2016). Readiness-to-change as a moderator of a web-based brief intervention for marijuana among students identified by health center screening. Drug Alcohol Depend.

[25] Saitz R, Palfai TP, Cheng DM, Horton NJ, Dukes K, Kraemer KL, Roberts MS, Guerriero RT, Samet JH (2009). Some medical inpatients with unhealthy alcohol use may benefit from brief intervention. J Stud Alcohol Drugs.

[26] Sherman BJ, Baker NL, McRae-Clark AL (2016). Gender differences in cannabis use disorder treatment: change readiness and taking steps predict worse cannabis outcomes for women. Addict Behav.

[27] Walton M, Goldstein A, Chermack S, McCammon R, Cunningham R, Barry K, Blow F (2008). Brief alcohol intervention in the emergency department: moderators of effectiveness. J Stud Alcohol Drugs.

[28] McConnaughy E, Prochaska J, Velicer W (1983). Stages of change in psychotherapy: measurement and sample profiles. Psychother Theor Res Pract.

[29] McConnaughy E, DiClemente C, Prochaska J, Velicer W (1989). Stages of change in psychotherapy: a follow-up report. Psychother Theor Res Pract.

[30] Boswell J, Sauer S, Gallagher M, Delgado N, Barlow D (2012). Readiness to change as a moderator of outcome in transdiagnostic treatment. Psychother Res.

[31] Dozois DJA, Westra HA, Collins KA, Fung TS, Garry JKF (2004). Stages of change in anxiety: psychometric properties of the University of Rhode Island Change Assessment (URICA) scale. Behav Res Ther.

[32] Litt MD, Shafer DM, Kreutzer DL (2010). Brief cognitive-behavioral treatment for TMD pain: long-term outcomes and moderators of treatment. Pain.

[33] Barnett NP, Apodaca TR, Magill M, Colby SM, Gwaltney C, Rohsenow DJ, Monti PM (2010). Moderators and mediators of two brief interventions for alcohol in the emergency department. Addiction.

[34] Freyer-Adam J, Baumann S, Schnuerer I, Haberecht K, Bischof G, John U, Gaertner B (2014). Does stage tailoring matter in brief alcohol interventions for job-seekers? A randomized controlled trial. Addiction.

[35] Grossbard J, Mastroleo N, Geisner I, Atkins D, Ray AE, Kilmer J, Mallett K, Larimer M, Turrisi R (2016). Drinking norms, readiness to change, and gender as moderators of a combined alcohol intervention for first-year college students. Addict Behav.

[36] Kelly A, Zuroff D, Foa C, Paul GP (2010). Who benefits from training in self-compassionate self-regulation? A study of smoking reduction. J Soc Clin Psychol.

[37] Lynch K, Van Horn D, Drapkin M, Ivey M, Coviello D, McKay JR (2010). Moderators of response to telephone continuing care for alcoholism. Am J Health Behav.

[38] Mastroleo NR, Murphy J, Colby SM, Monti PM, Barnett NP (2011). Incident-specific and individual level moderators of brief intervention effects with mandated college students. Psychol Addict Behav.

[39] Ouimet M, Dongier M, Di Leo I, Legault L, Tremblay J, Chanut F, Brown T (2013). A randomized controlled trial of brief motivational interviewing in impaired driving recidivists: a 5-year follow-up of traffic offenses and crashes. Alcohol Clin Exp Res.

[40] Project MATCH Research Group (1997). Matching alcoholism treatments to client heterogeneity: project MATCH Posttreatment drinking outcomes. J Stud Alcohol.

[41] Stice E, Marti N, Shaw H, O’Neil K (2008). General and program-specific moderators of two eating disorder prevention programs. Int J Eat Disord.

[42] Derogatis LR (1993). The brief symptom inventory (BSI): administration, scoring and procedures manual.

[43] Franke GH (2000). Brief symptom inventory (BSI).

[44] Margraf J (1994). MiniDIPS: Diagnostisches Kurz-Interview bei psychischen Störungen [MiniDIPS: Short diagnostic interview for mental disorders].

[45] DiNardo PA, Barlow DH (1988). Anxiety disorders interview schedule - revised (ADIS-R).

[46] American Society of Anesthesiologists (1963). New classification of physical status. Anesthesiology.

[47] Wolters U, Wolf T, Stuetzer H, Schröder T (1996). ASA classification and perioperative variables as predictors of postoperative outcome. Br J Anaesth.

[48] Charlson ME, Pompei P, Ales K, MacKenzie CR (1987). A new method of classifying prognostic comorbidity in longitudinal studies: development and validation. J Chronic Dis.

[49] Copeland GP, Jones D, Walters M (1991). POSSUM: a scoring system for surgical audit. Br J Surg.

[50] Noordzij PG, Poldermans D, Schouten O, Bax JJ, Schreiner FAG, Boersma E (2010). Postoperative mortality in the Netherlands: a population-based analysis of surgery-specific risk in adults. Anesthesiology.

[51] World Health Organization (1998). Info package: mastering depression in primary care, version 2.2.

[52] Kroenke K, Spitzer RL, Williams JB, Monahan PO, Loewe B (2009). An ultra-brief screening scale for anxiety and depression: the PHQ–4. Psychosomatics.

[53] Zigmond A, Snaith R (1983). The hospital anxiety and depression scale. Acta Psychiatr Scand.

[54] Reinert D, Allen J (2007). The alcohol use disorders identification test: an update of research findings. Alcohol Clin Exp Res.

[55] Hautzinger M (2003). Kognitive Verhaltenstherapie bei Depressionen, 6. Auflage [cognitive behavioural therapy for depression, 6th edition].

[56] Margraf J, Schneider S (1990). Panik: Angstanfälle und ihre Behandlung [panic disorders: anxiety attacks and their treatment].

[57] Becker E, Hoyer J (2005). Generalisierte Angststörung, Fortschritte der Psychotherapie, 25 [Generalized Anxiety Disorder, Progress in Psychotherapy, 25].

[58] Stangier U, Heidenreich T, Peitz M (2003). Soziale Phobien: ein kognitiv-verhaltenstherapeutisches Behandlungsmanual [social anxiety disorder: a cognitive behavioural therapy manual].

[59] Lindenmeyer J (2005). Alkoholabhängigkeit, Fortschritte der Psychotherapie, 6 [Alcohol dependence, Progress in Psychotherapy, 6].

[60] Graham HL (2004). Cognitive-behavioural integrated treatment (C-BIT): a treatment manual for substance misuse in people with severe mental health problems.

[61] Bundeszentrale für gesundheitliche Aufklärung [Federal Centre for Health Education] (2006). Leitfaden zur Kurzintervention bei Raucherinnen und Rauchern [Guideline for brief interventions for smokers].

[62] Heidenreich T, Hoyer J, Fecht J, Gloeckner-Rist A, Rist F, Kuefner H (2003). Veränderungsstadien-Skala (VSS) [University of Rhode Island Change Assesment, German version]. Elektronisches Handbuch zu Erhebungsinstrumenten im Suchtbereich (EHES) version 30 [electronic manual of assessment tools in the addiction field].

[63] Hoyer J, Heidenreich T, Fecht J, Lauterbach W, Schneider R (2003). Stadien der Veränderung in der stationären Alkoholentwöhnungstherapie [stages of change in alcohol inpatient treatment]. Verhaltenstherapie.

[64] Derogatis LR, Melisaratos N (1983). The brief symptom inventory (BSI): an introductory report. Psychol Med.

[65] Linnen H, Krampe H, Neumann T, Weiss-Gerlach E, Heinz A, Wernecke K-D, Spies CD (2011). Depression and essential health-risk factors in surgical patients in the preoperative anesthesiological assessment clinic. Eur J Anaesthesiol.

[66] Kerper LF, Spies CD, Lößner M, Salz A-L, Tafelski S, Balzer F, Weiß-Gerlach E, Neumann T, Lau A, Glaesmer H, Brähler E, Krampe H (2012). Persistence of psychological distress in surgical patients with interest in psychotherapy: results of a 6-month follow-up. PLoS One.

[67] Kerper LF, Spies CD, Buspavanich P, Balzer F, Salz A-L, Tafelski S, Lau A, Weiß-Gerlach E, Neumann T, Glaesmer H, Wernecke K, Brähler E, Krampe H (2014). Preoperative depression and hospital length of stay in surgical patients. Minerva Anestesiol.

[68] Hayes AF (2013). Introduction to mediation, moderation, and conditional process analysis: a regression-based approach.

[69] Hayes AF, Rockwood NJ. Regression-based statistical mediation and moderation analysis in clinical research: Observations, recommendations, and implementation. Behav Res Ther 2016;epub ahead of print: 10.1016/j.brat.2016.11.001. 10.1016/j.brat.2016.11.001 27865431

[70] IBM Corp. Released 2015. IBM SPSS statistics for windows, version 23.0. Armonk, NY: IBM Corp.

[71] Bohart AC, Greaves WA, Lambert MJ (2013). The client in psychotherapy. Bergin and Garfield’s handbook of psychotherapy and behavior change.

[72] Moyers TB, Miller WR, Hendrickson SM (2005). How does motivational interviewing work? Therapist interpersonal skill predicts client involvement within motivational interviewing sessions. J Consult Clin Psychol.

[73] Moyers TB, Rowell LN, Manuel JK, Ernst D, Houck JM (2016). The motivational interviewing treatment integrity code (MITI 4): rationale, preliminary reliability and validity. J Subst Abus Treat.

[74] Young J, Beck A (1980). Cognitive therapy scale: rating manual.

[75] Keeley RD, Brody DS, Engel M, Burke BL, Nordstrom K, Moralez E, Dickinson LM, Emsermann C (2016). Motivational interviewing improves depression outcome in primary care: a cluster randomized trial. J Consult Clin Psychol.

[76] Romano M, Peters L (2015). Evaluating the mechanisms of change in motivatinal interviewing in the treatment of mental health problems: a review and meta-analysis. Clin Psychol Rev.

[77] Westra HA, Constantino MJ, Antony MM (2016). Integrating motivational interviewing with cognitive-behavioral therapy for severe generalized anxiety disorder: an allegiance-controlled randomized clinical trial. J Consult Clin Psychol.

